# Praziquantel Treatment Decreases *Schistosoma mansoni* Genetic Diversity in Experimental Infections

**DOI:** 10.1371/journal.pntd.0002596

**Published:** 2013-12-19

**Authors:** Regina Coeli, Elio H. Baba, Neusa Araujo, Paulo M. Z. Coelho, Guilherme Oliveira

**Affiliations:** 1 Genomics and Computational Biology Group, Centro de Pesquisas René Rachou, Fiocruz, Belo Horizonte, Minas Gerais, Brazil; 2 Laboratory of Schistosomiasis, Centro de Pesquisas René Rachou, Fiocruz, Belo Horizonte, Minas Gerais, Brazil; 3 National Institute of Science and Technology in Tropical Diseases, Centro de Pesquisas René Rachou, Fiocruz, Belo Horizonte, Minas Gerais, Brazil; University of Nottingham, United Kingdom

## Abstract

**Background:**

Schistosomiasis has a considerable impact on public health in many tropical and subtropical areas. In the new world, schistosomiasis is caused by the digenetic trematode *Schistosoma mansoni*. Chemotherapy is the main measure for controlling schistosomiasis, and the current drug of choice for treatment is praziquantel (PZQ). Although PZQ is efficient and safe, its repetitive large-scale use in endemic areas may lead to the selection of resistant strains. Isolates less susceptible to PZQ have been found in the field and selected for in the laboratory. The impact of selecting strains with a decreased susceptibility phenotype on disease dynamics and parasite population genetics is not fully understood. This study addresses the impact of PZQ pressure on the genetics of a laboratory population by analyzing frequency variations of polymorphic genetic markers.

**Methodology:**

Infected mice were treated with increasing PZQ doses until the highest dose of 3×300 mg/Kg was reached. The effect of PZQ treatment on the parasite population was assessed using five polymorphic microsatellite markers. Parasitological and genetic data were compared with those of the untreated control. After six parasite generations submitted to treatment, it was possible to obtain a *S. mansoni* population with decreased susceptibility to PZQ. In our experiments we also observed that female worms were more susceptible to PZQ than male worms.

**Conclusions:**

The selective pressure exerted by PZQ led to decreased genetic variability in *S. mansoni* and increased endogamy. The understanding of how *S. mansoni* populations respond to successive drug pressure has important implications on the appearance and maintenance of a PZQ resistance phenotype in endemic regions.

## Author Summary

Schistosomiasis causes severe health problems and economic losses in transmission areas in the Americas, Africa, and Southeast Asia. In regions where treatment is available, such as Brazil, chemotherapy has led to decreased morbidity of the disease. However, parasite resistance to drugs is of general concern. Drug resistance is known in some species of helminths, and there are reports of decreased susceptibility in schistosomes, although such reports are not widespread. The impact of consecutive drug treatment on parasite populations at transmission sites can be evaluated by the genetic makeup of the population. For example, the decrease in the genetic diversity of these populations upon chemotherapy can be interpreted as the selection of less sensitive parasites. In this study, we used a laboratory mouse model of schistosomiasis that was treated with increasing amounts of praziquantel (the drug of choice for human treatment) until we reached the highest dose of 3×300 mg PZQ/kg. Our results clearly indicate that the parasite population showed a decrease in genetic diversity after measuring allelic and genetic diversity. This observation confirms that drug selection occurred in this population. The work highlights the need for genetic monitoring of populations under drug pressure, resistance mechanisms, and the dynamics of parasite populations upon treatment.

## Introduction

Schistosomiasis is a disease that affects approximately 206 million people in 76 countries. On a global scale, 779 million people are at risk of contracting the disease [1]. Currently, the main approaches for the reduction of schistosomiasis morbidity are the treatment of infected patients and sanitation [2]. The life cycle of the parasite is complex, involving a vertebrate host and a snail vector. Infection occurs when parasites in the infective stage (cercariae) are released from aquatic snails and penetrate the skin or mucosa of the vertebrate host upon contact with water. By migrating through the circulatory system, the larvae (schistosomula) reach the portal hepatic vein where they mature into adult worms and migrate to the mesenteric veins. The female then deposits eggs in the intestinal vein system. Some eggs pass through the gut lumen and are eliminated with the feces, or urine in the case of *S. haematobium*, while others are trapped in the host tissues where they produce a granulomatuous immune-inflammatory process that represents the main cause of pathology [3].

Praziquantel (PZQ) is the drug used for treatment of human schistosomiasis and is inexpensive and effective against all human schistosome and some cestode species [4]. However, PZQ is less effective against the immature stages of *Schistosoma mansoni* [5]. PZQ appears to exert multiple effects on schistosomes, damaging the tegument and causing muscle contraction [6]. These morphological alterations are accompanied by an increased exposure of parasite antigens on the worm surface, which in turn appears to render the worms more susceptible to attack by the immune system [7]. The relationship between PZQ and Ca^+2^ influxes suggests that the sites of action are Ca^2+^ ion channels in the membrane of tegument and muscle cells [8], [9], [10]. Other effects of PZQ in *Schistosoma* that have been demonstrated include the following: reduction in the level of glutathione (GSH) [11]; inhibition of the male excretory system [12]; inhibition of nucleoside uptake [13]; and increase in MDR transporter activity [14], [15]. However, a complete description of PZQ's mode of action has not yet been accomplished.

The availability of only one drug to treat schistosomiasis is a concern, and too little investment has been made toward the development of alternatives [16]. There is some natural variation in the parasite sensitivity to PZQ. The variability of *S. mansoni* strains regarding susceptibility to schistosomicidal drugs was demonstrated in different endemic regions, such as Senegal, Egypt and Brazil [17], [18], [19], [20], [21], [22], [23]. It is conceivable that in an endemic situation a resistant phenotype may be selected for under frequent drug pressure [18]. Resistant, less susceptible isolates or parasites that survive treatment of *S. mansoni* in endemic regions may emerge by different mechanisms. The main causes may be high parasitic loads, fast reinfection, the presence of immature worms, inefficient human immune responses, the use of low quality drugs, differences in evaluation, and cure criteria or actual drug resistance [24], [25].

The first study that showed it is possible to induce S. *mansoni* less susceptible to PZQ was published by Fallon and Doenhoff [26]. Recently, PZQ resistant populations were also obtained after the intramolluscan parasite phase was exposed to the drug [27]. The genetics of *S. mansoni* populations are being investigated with the use of highly polymorphic microsatellite markers [28]; the availability of a genetic MAP significantly contributed to these efforts [29]. Polymorphic microsatellites have proven to be extremely useful in describing the genetic structure of field and laboratory isolates [30], [31], [32], [33], [34]. However, little is known about the genetics of PZQ susceptibility [35], [36].

Understanding how drug pressure affects the genetic structure of a parasite population in a controlled setting raised questions relevant to the endemic setting related to transmission, chemotherapy effects, and epidemiology of the disease [35], [36], [37]. Studies of how *Schistosoma* populations at endemic sites perform under drug pressure are necessary for determining the most efficient treatment strategy for infected populations and for the design and modeling of control programs [38], [39]. Because PZQ is the drug of choice being used in the population control of schistosomiasis, studies about the genetics of populations under drug pressure and new drug targets are urgently needed. In this paper, we hypothesize that in a mouse system, as a result of a regimen of exposures to increasing amounts of PZQ, the *S. mansoni* population will experience a decrease in genetic diversity and susceptibility to the drug.

## Materials and Methods

### Ethics statement

*In vivo* studies were conducted in compliance with the guidelines of the Collegiate of Animal Experimentation (COBEA) and approved by the Commission on the Ethical Use of Animals (CEUA-FIOCRUZ) protocol number L-018/09 approved on Jan. 23, 2009.

### Parasites and hosts

The *S. mansoni* (LE strain) life cycle was maintained using *Biomphalaria glabrata* snails as intermediate hosts and Swiss mice as definitive hosts according to Pellegrino and Katz [40]. Female albino mice weighing approximately 20 g were infected with 100±10 cercariae of *S. mansoni* (LE strain) by a subcutaneous route.

### Induction of decreased susceptibility to PZQ

To induce decreased susceptibility, increasing doses of an aqueous suspension of PZQ were administered to infected mice by oral gavage. The protocol used for parasite selection was based on Fallon and Doenhoff [26] with one modification: in the third treatment, mice were given two doses of 200 mg/kg. The dose of 200 mg/kg PZQ was used to ensure that some of the parasites survived drug treatment for subsequent passages until the highest dose of 3×300 mg/kg was reached. The first and second rounds of treatment started with two subsequent doses of 100 mg/kg on days 28 and 35 after infection. The eggs produced by the worms that survived drug treatment were used to infect snails, which, after 35 days, started to eliminate cercariae that were used to infect mice. For the third and fourth rounds of treatment, subsequent doses of 2×200 mg/kg on days 28 and 35 were used. At the fifth treatment round, mice were treated with two doses of 250 mg/kg and after the sixth round, treatment was maintained with three doses of 300 mg/kg on days 28, 35 and 37 until the treatment rounds were completed. Five weeks after each round of PZQ treatment the mice were portally perfused and the surviving worms recovered were counted. The worm population obtained after seven successive treatment rounds was considered the Selected population (S). For each round of PZQ treatment the same procedure was used in a group of mice infected with cercariae produced by snails from the Non-selected population (N). Adult worms in the Selected and Non-selected groups were retrieved by perfusion of the portal system with 0.85% saline solution and 1% heparin [41]. Four groups of 20 mice were used for each cycle. Two groups (Selected and Non-selected populations) were left untreated and animals were portally perfused 45 days after infection to obtain an infection control worms count. The other two groups were treated as described above and mice were sacrificed five weeks after treatment.

### DNA extraction

For DNA extraction of individual parasites, worm pairs were separated and digested in extraction buffer (50 mM Tris-HCl pH 8.0, 100 mM EDTA, 100 mM NaCl and 0.5% SDS) and 20 µg/ml of proteinase K (Sigma) and incubated for 12 hours at 37°C, followed by incubation at 95°C for 5 min. Nucleic acids were precipitated with cold absolute ethanol and resuspended in 30 µl of TE buffer and rehydrated for 12 hours at 4°C.

### Amplification of microsatellite loci

We analyzed five polymorphic microsatellite *loci* for each population. Allelic and genotypic data were identified for a total of 250 worms. PCR reactions and scoring of the polymorphic microsatellites were conducted as previously described [42], [43]. Primer pairs and genomic locations are described in Table 1.

**Table 1:** Primers used for microsatellite genotyping, and genomic localization.

Primers	Sequence	Genome Localization	Gene	Reference
SmBr5	For: GAATTACTGTCCCTTTATCTC	Smp_scaff000422	Smp-171310 gene	
	Rev: AAACTATTCATTACTGTCGGG	Location: 224437–224454	Hypothetical protein	42
SmBr6	For: CTTAACAGACATACACGC	Smp_scaff000003	Intergenic region	42
	Rev: GAATACAGGCTATAATCTACA	Location: 1550379–1550637		
13TAGA	For: GTACATTTTATGTCAGTTAGCC	Smp_scaff000419	Smp_089460 gene	43
	Rev: CATGATCTTAGCTCAGAGAGC	Location: 158262–158282	Calpain B, putative	
SmBr9	For: ATTCACCCATTGTCTTAAAACC	Smp_scaff000316	Intergenic region	43
	Rev: ATTGGCGTCAGTAGAAGAGATT	Location: 14607–14767		
SmBr13	For: GTCACAGATACCTGACGAGCTG	Smp_scaff000328	Intergenic region	43
	Rev: ACTCCCCAGCAATTTGTCC	Location: 185390–185611		

The genomic localization of microsatellites was identified using Blast Search in SchistoDB [54].

### Genetic analysis

Genetic data was analyzed with the Fstat software version 2.9.3.2 [44]. For each locus, allelic and genotypic frequencies, linkage disequilibrium among polymorphic loci, the inbreeding coefficient, and observed and expected heterozygosity values were calculated. Hardy-Weinberg (HW) equilibrium was calculated using the Arlequin software [45]. Population structure was estimated for the isolates using the fixation indices Fst [46], Fis [47] and Rst [48]. The Guo and Thompson test [49] was used to evaluate significance of deviations of the observed and expected heterozygosity between Selected and Non-selected worms and to assess the difference between genetic variability of male and female worms (P≤0.05). Statistical analysis of parasitological data was conducted with the SPSS software version 11. The mean number of worms recovered after selection or lack of selection by PZQ was compared using the Mann-Whitney and Kruskall-Wallis tests. To evaluate the significance between female and male worm burdens, the Wilcoxon test was used. P-values≤0.05 were considered to be statistically significant. The percent reduction of worm burden in each treatment group was calculated according Cioli et al. [23]. Approximately 200 worms each from treated and untreated mice were used to produce the genetic data.

## Results and Discussion

### Parasitological data

PZQ selection resulted in a striking increase in the number of recovered parasites after treatment with this drug, compared with the number recovered after equivalent treatment of non-selected parasites. Thus, after seven parasite generations of selection using doses of PZQ that increased from 200 mg/kg to 900 mg/kg, we observed a greater than three-fold increase in the degree to which the proportion of worm load in the Non-selected group had been reduced by drug treatment (47.3%), compared with the level of reduction in the Selected group (14.58% - Table 2). These findings are in agreement with previous observation of laboratory selection reported by Fallon and Doenhoff [26].

**Table 2:** Worms recovered and the percentage of worm reduction after PZQ pressure.

Passage	Treatment	Mean number of worms ± SD (% reduction)	
	(mg/kg of PZQ)	Non-selected	Selected
1	Untreated	32.15±9.66	Υ
	2×100 mg	27.84±11.03 (13.42)	27.84±11.03 (13.42)
6	Untreated	42.90±14.10	17.88±7.63
	3×300 mg	17.22±10.59 (59.88)*	14.00±8.78 (21.68)*
7	Untreated	77.22±12.32	67.83±18.33
	3×300 mg	40.69±14.36 (47.30)**	57.94±12.09 (14.58)**

*, ** p<0.05 (Non-selected vs. Selected). N = 20 mice were used for each group.

^Υ^The initial control group is the same for the Non-selected and Selected populations.

Mean numbers of worms recovered from treated and untreated mice after the first, sixth and seventh rounds of PZQ treatment. % Percentage of reduction of Selected (S) and Non-selected (N) worms obtained after treatment with PZQ at 2×100 mg/kg and 3×300 mg/kg. For each round, eggs produced by the worms that had survived the drug treatment were used to infected snails, and the cercariae obtained were used to infect a new set of mice.

*, ** P≤0.05 indicates a significant difference between Selected and Non-selected parasites in the percent worm reduction upon PZQ treatment (Mann Whitney).

SD: standard deviation.

Female adult worms were shown to be more susceptible than males to PZQ exposure. We observed that after exposure to 3×300 mg/kg of PZQ, both male and female worms showed a significant decrease in susceptibility. Table 3 shows the results obtained after treatment of infected mice with 3×300 mg/kg doses of PZQ after 11 passages, indicating the increased sensitivity of female adult worms in comparison to male worms. After treatment, the male to female ratio changed from 2.5 in the control population to 8.7 in the Selected population. A decreased recovery of males was observed by Delgado et al. [50]. It has been observed that tegumental damage induced by PZQ is more apparent in male worms, while the female tegument displays limited damage [51]. Pica-Mattoccia and Cioli [52] observed that immature females are refractory to PZQ treatment, but upon sexual maturation they become sensitive to PZQ. The same researchers showed that *in vitro* males displayed higher sensitivity to PZQ. These experiments differ from ours in that we assessed PZQ sensitivity *in vivo*. It has been suggested that the environment in which the parasite is located affects the parasite physiology and drug sensitivity [52]. This is especially relevant for females living in the gynecophoric canal of males, where they may be more protected from PZQ activity. It has also been reported that resistance can lead to decreased reproductive fitness in schistosomes [53], [38]. Previous laboratory studies show that selection can rapidly change infectivity, virulence phenotypes, and population structure of schistosomes after a few generations [34]. The decrease in the female population was so striking after 11 passages that it resulted in the impossibility to maintain the strain in the laboratory. Our study shows that in a short time PZQ can select against female parasites, and such variations may substantially influence transmission dynamics, thereby decreasing the spread of decreased susceptibility to PZQ.

**Table 3:** Ratio of male/female adult worms recovered from the Non-selected and Selected populations after 11 passages.

		Non-selected		Selected	
		Ratio	Average (SD)	Ratio	Average (SD)
Untreated	Male	2.4	18.6 (6.9)	1.8	13.6 (4.9)
	Female		7.7 (4.4)		7.5 (4.6)
Treated	Male	2.5	6.5 (3.7)	8.7	11.3 (3.9)
	Female		2.6 (2.5)		1.3 (1.2)

Treatment was carried out with 3×300 mg/kg of PZQ. Ratio between male or female parasites recovered after treatment of the Non-selected and Selected populations and control populations. Average number of male and female worms recovered after treatment in the Non-selected and Selected populations. SD: standard deviation.

### Genetic data

PZQ-mediated selection led to decreased allelic diversity in *S. mansoni*. The number of alleles found in the Non-selected population decreased in the Selected population from 98 to 42. The number of genotypes found in the Non-selected population and the Selected population decreased from 265 to 112. The allele and genotype number for each locus decreased after successive treatments. The Selected population (S) showed a lower total number of alleles per genotype locus than that of Non-selected parasites (Table 4). It was clearly demonstrated that the selection process decreased parasite genetic diversity.

**Table 4:** Allele and Genotype numbers in Non-selected (N) and Selected (S) populations.

Population	Locus	Number of worms	Number of alleles	Number of genotypes
N	Smbr5	202	19	52
	Smbr6	248	20	65
	Smbr9	211	19	40
	Smb13	213	14	36
	13TAGA	199	26	72
			Total 98	Total 265
S	Smbr5	203	7	28
	Smbr6	247	7	33
	Smbr9	211	7	46
	Smb13	214	7	37
	13TAGA	199	14	33
			Total 42	Total 112

Number of adult worms tested, in the Non-selected (N) and Selected (S) populations after 7 passages. The number of alleles and genotypes was determined by PCR. Scoring of the polymorphic microsatellite was conducted as described in Materials and Methods by using specific primers for each microsatellite locus. PCR reactions were performed in S and N worms obtained as described in the legend of Table 2.

The degrees of population structuring and diversity among Selected and Non-selected populations were evaluated by genetic diversity analysis (R*st*) and Inbreeding Coefficient (F*is*). Our results show that there is a significant genetic differentiation between the first and seventh generations of the S population (R*st* = 0.240). In contrast, without PZQ pressure, we did not observe a significant differentiation in the Non-selected populations (R*st* = 0.080). Genetic diversity data reveal that decreased susceptibility selection contributes to the decrease of genetic variability of parasites selected with PZQ. Next, we measured the level of inbreeding between the Selected and Non-selected populations. According to Wright [47], a F*is* value of 0.309 indicates a high level of inbreeding in the Selected population. The F*is* index shows that there is a strong increase in endogamy in the Selected population (0.134 to 0.309). Our results with both indexes suggest that the Selected *S. mansoni* population is less genetically diverse as a result of PZQ pressure. These results agree with a report by Norton and colleagues [36], which showed that in an endemic setting a single round of mass drug administration to a human population produced a genetic bottleneck on *S. mansoni*. We did not observe significant differences in the genetic variability of male and female worms (data not shown) nor in populations passaged in parallel but not submitted to drug pressure.

The analysis of Hardy-Weinberg equilibrium deviation is an important resource for the study of the evolutionary mechanisms that can act upon a given population. The comparisons between the expected and observed heterozygosities in Selected and Non-selected populations show that the observed heterozygosity was lower than expected for all polymorphic loci except SmBr 9 in the N strain (Table 5). HW equilibrium deviation occurred in the majority of analyzed loci along with the loss of observed heterozygosity in the S and N populations. The analysis of the genetic structure of the N and S populations using the Hardy-Weinberg coefficient agrees with the genetic diversity index and endogamy coefficient and indicates decreased genetic diversity in the population under drug pressure. The maintenance of the *S. mansoni* LE strain by PZQ-mediated selective pressure contributed to the decrease in variability of the S population. The genetic bottleneck and subsequent loss in genetic diversity were also observed in parasites found at endemic sites that had received PZQ treatment [36]. One explanation for the observed deficiency in heterozygosity in the Selected population could be selective pressures suffered by this strain after seven generations under drug pressure [39]. Taken together, these results may indicate that at endemic sites, there may be a larger pool from which genetically resistant strains may be positively selected under drug pressure.

**Table 5:** Expected and observed heterozygosity in the Selected and Non-selected populations.

Population	Smbr6		Smbr5		Smbr13		Smbr9		13TAGA	
	Ho	He	Ho	He	Ho	He	Ho	He	Ho	He
N	0.43	0.67*	0.69	0.77*	0.86	0.70*	0.78	0.62	0.73	0.78*
S	0.71	0.75*	0.20	0.79*	0.65	0.71*	0.62	0.79*	0.65	0.87*

*Guo and Thompson test p≤0.05.

Microsatellite markers were tested for expected (He) and observed (Ho) heterozyzosity in Non-selected (N) and Selected (S) populations.

In this paper, we clearly demonstrate the effects of PZQ selection on populations displaying decreased susceptibility to PZQ. We observed a decrease in the genetic diversity of the parasite. In addition, the apparent cost of coping with large doses of PZQ is an imbalance in the male/female ratio. In the laboratory, the consequence was the difficulty of maintaining the strain. Although PZQ may cause changes in the genetic structure of treated populations, our results suggest a slow spread of drug resistance because after 11 rounds of treatment we recovered almost exclusively female worms. Levels of genetic diversity, as opposed to genetic divergence between populations, may itself be an important component of the epidemiology of infection and disease, as well as a key indicator for monitoring the effects of selection imposed by drug treatment (36). We speculate that in the field, one possible reason for resistance or decreased susceptibility not being widespread is perhaps its biological cost. The creation of a genetic map constructed using microsatellites, combined with the future development of a SNP map, means that genetic tools will be invaluable in both investigating the genetic profile of populations and identifying markers or genes that convey resistance.
